# Sensitive biomarkers of alcoholism's effect on brain macrostructure: similarities and differences between France and the United States

**DOI:** 10.3389/fnhum.2015.00354

**Published:** 2015-06-23

**Authors:** Anne-Pascale Le Berre, Anne-Lise Pitel, Sandra Chanraud, Hélène Beaunieux, Francis Eustache, Jean-Luc Martinot, Michel Reynaud, Catherine Martelli, Torsten Rohlfing, Adolf Pfefferbaum, Edith V. Sullivan

**Affiliations:** ^1^Department of Psychiatry and Behavioral Sciences, Stanford University School of MedicineStanford, CA, USA; ^2^INSERM, EPHE, Université de Caen/Basse-Normandie, Unité U1077, GIP Cyceron, CHU de CaenCaen, France; ^3^EPHEBordeaux, France; ^4^INCIA, UMR-Centre National de la Recherche Scientifique 5287, Université BordeauxBordeaux, France; ^5^Neuroscience Program, SRI InternationalMenlo Park, CA, USA; ^6^INSERM Research Unit 1000, Université Paris-Sud et Université Paris Descartes, SHFJ, I2BMOrsay, France; ^7^INSERM, UMR 669Villejuif, France; ^8^Department of Psychiatry and Addictology, APHP, Paul Brousse HospitalVillejuif, France; ^9^Faculté de Médecine, Université Paris-Sud 11Le Kremlin Bicêtre, France

**Keywords:** alcoholism, brain, cerebrospinal fluid, France, Korsakoff syndrome, MRI, United States, white matter

## Abstract

Alcohol consumption patterns and recognition of health outcomes related to hazardous drinking vary widely internationally, raising the question whether these national differences are reflected in brain damage observed in alcoholism. This retrospective analysis assessed variability of alcoholism's effects on brain cerebrospinal fluid (CSF) and white matter volumes between France and the United States (U.S.). MRI data from two French sites (Caen and Orsay) and a U.S. laboratory (SRI/Stanford University) were acquired on 1.5T imaging systems in 287 controls, 165 uncomplicated alcoholics (ALC), and 26 alcoholics with Korsakoff's Syndrome (KS). All data were analyzed at the U.S. site using atlas-based parcellation. Results revealed graded CSF volume enlargement from ALC to KS and white matter volume deficits in KS only. In ALC from France but not the U.S., CSF and white matter volumes correlated with lifetime alcohol consumption, alcoholism duration, and length of sobriety. MRI highlighted CSF volume enlargement in both ALC and KS, serving as a basis for an *ex vacuo* process to explain correlated gray matter shrinkage. By contrast, MRI provided a sensitive *in vivo* biomarker of white matter volume shrinkage in KS only, suggesting a specific process sensitive to mechanisms contributing to Wernicke's encephalopathy, the precursor of KS. Identified structural brain abnormalities may provide biomarkers underlying alcoholism's heterogeneity in and among nations and suggest a substrate of gray matter tissue shrinkage. Proposed are hypotheses for national differences in interpreting whether the severity of sequelae observe a graded phenomenon or a continuum from uncomplicated alcoholism to alcoholism complicated by KS.

## Introduction

According to the Global Status Report on Alcohol and Health (2014) published by the World Health Organization (WHO) [http://www.who.int/substance_abuse/publications/global_alcohol_report/en/], alcohol consumption amount, patterns, and health outcomes vary widely by country. Total alcohol consumption per capita (age 15 years and over) in France (10.0–12.4 liters with 56% wine drinkers) is higher than in the United States (U.S.) (7.5–9.5 liters with 50% beer drinkers), as is the prevalence of heavy episodic (binge) drinking. Nonetheless, both countries have a similar estimated pattern of drinking scores assessing four different dimensions of heavy drinking occasions (daily drinking, frequency of getting drunk, usual quantity per drinking session, holiday binge drinking), drinking with meals, and drinking in public places (Rehm et al., 2003). Taken together, these drinking indices provide estimates of risk and disease burden. Nations also vary in recognition of alcoholism as a problem, evidenced by the existence of weak written national alcohol policies and prevention programs in a large proportion of countries. While both the U.S. and France adopted written national policies, France, unlike the U.S., has no national action plan. France also has a lower legal minimum age for off/on-premise sales of alcoholic beverages (18 years old) than the U.S. (21 years old), but stricter maximum legal blood alcohol concentration when driving a vehicle (0.05%) compared with the U.S. (0.08%). National and multi-national studies on the untoward effects of hazardous drinking have the potential of raising awareness, identifying sources of alcohol-related problems, and indicating areas to target in reversing or ameliorating resulting problems.

For two generations, neuroimaging studies have identified consistent biomarkers of alcohol dependence. Because of resolution and tissue conspicuity limitations, early CT studies focused on fluid-filled spaces of the ventricular system and cortical sulci (Jernigan et al., 1982; Pfefferbaum et al., 1988) but were also successful in recognizing improvement in the condition of the brain with sustained sobriety (Carlen et al., 1978; Muuronen et al., 1989; Mann et al., 1993). Later-generation imaging with MRI enabled quantification of tissue compartments and indicated further shrinkage of white matter volumes with continued drinking and their enlargement with sobriety (Shear et al., 1994; Pfefferbaum et al., 1995; O'Neill et al., 2001; Agartz et al., 2003). Consistent across imaging modalities is variation of lateral ventricles with drinking and abstinence, a phenomenon replicated in controlled rodent models of alcoholism (Pfefferbaum et al., 2008; Zahr et al., 2013).

Korsakoff's Syndrome (KS) is a major neurological complication of chronic alcohol consumption characterized by a profound global amnesia (Kopelman, 1995; Pitel et al., 2008) ensuing from severe alcohol-related thiamine deficiency (Thomson, 2000). *In vivo* neuroimaging studies conducted in KS have revealed cortical thinning, Sylvian fissure and sulcal widening, and ventricular expansion (Shimamura et al., 1988; Jacobson and Lishman, 1990; Visser et al., 1999; Sullivan and Pfefferbaum, 2009). Widespread gray matter and white matter volume deficits in the nodes and connections of the frontocerebellar and limbic circuits in KS compared to controls have previously been demonstrated (Pitel et al., 2009), as well as graded effects of volume deficits in the genu of the corpus callosum from uncomplicated alcoholics to KS (Pitel et al., 2012).

Recognizing the salience of ventricular, cortical sulcal, and white matter volume measures as sensitive to the effects of chronic alcoholism, as well as the potential of their reversal with sobriety, this retrospective, multi-site, international study used a common quantification method to measure regional CSF and white matter volumes in 478 alcoholics and controls. Previous measurement of the gray matter nodes of the limbic and frontocerebellar systems in this international cohort revealed a common graded deficit across alcoholism severity based on presence or absence of KS. Yet, national differences were evident in selective subcortical volumes despite similar alcohol consumption levels in both countries, thus highlighting specific country-related patterns of regional brain shrinkage (Le Berre et al., 2014).

The current analysis of these imaging data focused on ventricular, cortical sulcal, and white matter volumes as common biomarkers of alcohol dependence and aimed at (1) quantifying regional white matter and CSF volume abnormalities with the hypothesis of graded effect by diagnostic severity, (2) identifying whether alcohol history variables and age account for potential national differences in white matter and CSF volumes, and (3) testing whether gray matter deficits identified previously (Le Berre et al., 2014) were commensurate with shrinkage of white matter or could be accounted for by expansion of CSF volumes, the latter suggesting an *ex vacuo* process. The hydrocephalus *ex vacuo* process refers to a compensatory and asymptomatic enlargement under normal pressure of both the cortical sulci and the ventricles in response to the shrinkage of surrounding brain tissue (Symonds et al., 1999).

## Methods

### Participants

Normal controls (NC), nonamnesic patients with alcohol-dependence (ALC), and patients with an alcoholic KS were recruited across two French sites (Caen and Orsay) and a U.S. laboratory (SRI/Stanford University). All 478 participants were included in a previous retrospective structural neuroimaging investigation focused on the heterogeneity of alcohol's effects on gray matter nodes of the frontocerebellar and limbic circuitry between France and the U.S. (Le Berre et al., 2014).

The French sample was composed of 112 participants: 11 patients with KS (all from Caen), 48 nonamnesic patients with ALC (23 from Caen and 25 from Orsay), and 53 NC (25 from Caen and 28 from Orsay). The U.S. sample comprised 366 participants: 15 patients with KS (10 included from a Veterans Administration (VA) Medical Center and 5 from SRI International), 117 nonamnesic patients with ALC (59 from VA and 58 from SRI), and 234 NC (187 from VA and 47 from SRI). The ALC patients were interviewed to determine drinking history variables such “duration of alcoholism” operationalized according to the age of onset of alcoholism, which is the age when a patient met for the first time the diagnostic criteria for alcohol dependence, “lifetime alcohol consumption,” which is the estimated quantity of alcohol consumed in kg since the patient started drinking alcohol in a regular way, and “length of sobriety,” which is the number of days of abstinence prior the inclusion in the study according to the date of last drink. The three diagnostic groups differed in age, and sites differed in sex ratio (Table 1). French ALC patients had a shorter duration of alcoholism than those in the U.S., whereas the groups did not differ in lifetime alcohol consumption or length of sobriety (Table 1). Patients from both countries were recruited from treatment programs.

**Table 1:** **Demographic data (mean ± S.D.) of the samples from France and the U.S. for the three diagnoses: normal control subjects (NC), patients with alcoholism (ALC), and patients with Korsakoff's Syndrome (KS)**.

	**FRANCE**			**U.S.**			**Effect of the site**		**Effect of the diagnosis**	
	**NC**	**ALC**	**KS**	**NC**	**ALC**	**KS**	**χ^2^-, *F*-, *t-value***	***p***	**χ^2^, *F-value***	***p***
*n*	53	48	11	234	117	15	*/*	*/*	*/*	*/*
Sex (women/men)	14/39	3/45	4/7	87/147	50/67	4/11	*14.97^a^*	< 0.001^*^	*0.56^a^*	*0.76*
Age (years)	51.83 ± 10.37	45.52 ± 8.10	52.91 ± 10.10	49.41 ± 16.79	45.60 ± 11.50	64.33 ± 10.78	*0.27^c^*	*0.78*	*12.74^b^*	<0.001^*^
										***KS > NC > ALC***
Lifetime alcohol consumption (kg)	/	1173.68 ± 1188.55	/	/	1000.42 ± 866.43	/	*1.04^c^*	*0.30*	*/*	*/*
Duration of alcoholism (years)	/	11.65 ± 9.69	/	/	20.09 ± 10.98	/	*−4.64^c^*	<0.001^*^	*/*	*/*
Alcohol abstinence prior to the study (days)	/	173.77 ± 487.20	/	/	300.45 ± 524.52	/	*−1.44^c^*	*0.15*	*/*	*/*

a *Pearson χ^2^*;

b *univariate ANOVA: F-value*;

c *independent samples T-test: t-value*.

* *Significant effect at p < 0.05 in bold and italic*.

DSM-IV criteria determined alcohol dependence (American Psychiatric Association, 1994) in the French group; either Research Diagnostic Criteria (RDC) for alcoholism (Spitzer et al., 1975) or DSM-IV criteria for alcohol dependence (American Psychiatric Association, 1994) were used for the U.S. group, depending on inclusion year. The Research Diagnostic Criteria for alcohol dependence (RDC), a forerunner of DSM criteria, demonstrated a high level of agreement with the DSM-III, with even stricter criteria for determining alcohol dependence than required by DSM (Leonard et al., 1984). According to family reports and medical records, all KS patients had a documented history of long-term heavy alcohol drinking and met criteria for DSM-IV Alcohol-Induced Persisting Amnestic Disorder (American Psychiatric Association, 1994). Their profound memory impairment precluded collection of reliable alcohol history. NC participants were non-drinkers or social drinkers as defined by the National Institute on Alcohol Abuse and Alcoholism.

Exclusion criteria applied to all participants: use of psychotropic medication; significant psychiatric or medical histories including head injury with loss of consciousness; coma; epilepsy; stroke; anxiety or depressive disorders; hepatic encephalopathy; substance dependence other than alcohol and nicotine in the ALC and KS groups; past or present alcohol or drug abuse or dependence in the NC group.

All participants gave their written informed consent before entry to the study, which was conducted in line with the Declaration of Helsinki; in a few cases of KS, a legal guardian also granted a written consent. The study was approved for Caen by the local ethics committee for human investigations (CPP Nord-Ouest III), for Orsay by the Bicètre ethics committee for human investigations in Orsay, and for the United States by the Institutional Review Boards (IRB) of Stanford University School of Medicine and the Veterans Administration, and for later study components by IRBs of Stanford University School of Medicine and SRI International.

### MRI data acquisition and preprocessing

All MRI data were T1-weighted (SPGR) sequences, acquired on General Electric whole-body magnet systems at the same magnet strength (1.5T). The different parameters in acquisition protocols among sites are listed in Table 2.

**Table 2:** **MRI acquisition protocol parameters in French sites (Caen and Orsay) and the U.S. sites (VA and SRI)**.

	**Caen**	**Orsay**	**U.S. VA**	**U.S. SRI**
Slice orientation	Axial	Axial	Sagittal	Coronal
Slice count	128	124	124	94
Slice thickness in mm	1.5	1.3	1.5	2.0
Repetition time (TR) in ms	10.3	10.0	24.0	25.0
Echo time (TE) in ms	2.1	2.0	5.0	5.0
In-plane resolution (mm)	0.9375 × 0.9375	0.9375 × 0.9375	0.9375 × 1.25	0.9375 × 1.25

As described previously (Le Berre et al., 2014), the data from the two countries were pooled, preprocessed and analyzed together at the U.S. site using a single, common quantification approach, an atlas-based parcellation procedure (Rohlfing et al., 2010).

#### Image preprocessing

All structural images were first corrected for intensity bias by applying a second-order polynomial multiplicative bias field computed via entropy minimization (Likar et al., 2001). The SPGR images were each skull stripped using FSL's Brain Extraction Tool, BET (Smith, 2002).

#### Registration, atlas-based parcellation, and tissue segmentation

For each subject, the skull-stripped SPGR image was registered to the SPGR channel of the SRI24 atlas (Rohlfing et al., 2010) (http://nitrc.org/projects/sri24) via nonrigid image registration (Rohlfing and Maurer, 2003). All bias-corrected and skull-stripped SPGR images were segmented into three tissue compartments (gray matter, white matter, CSF) using FMRIB's Automated Segmentation Tool (FAST) in FSL (Zhang et al., 2001). As tissue priors to both initialize and guide the classification, we used the tissue probability maps provided with the SRI24 atlas, reformatted into subject SPGR space via the transformations described above.

#### Regions of interest (ROIs)

**Figure 1:**
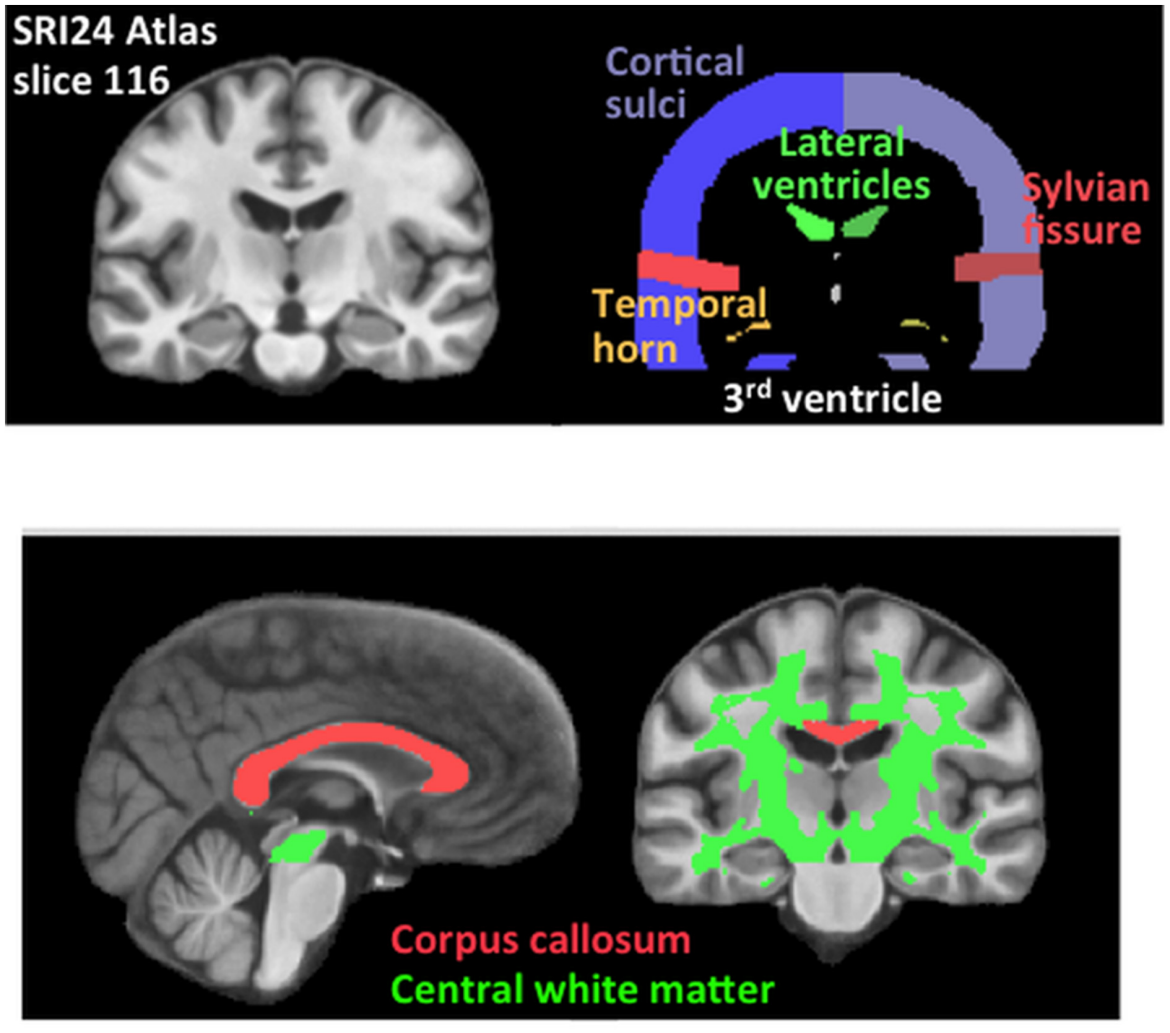
**Brain cerebrospinal fluid and white matter parcellated regions of interest (ROIs)**. Sagittal and coronal slices are from the SRI24 atlas, displaying color-coded ROIs used in the analyses.

CSF ROIs were the lateral and third ventricles, Sylvian fissures, and cortical sulci. White matter ROIs were the corpus callosum and a large volume of white matter subtending most of the centrum semiovale (Figure 1).

### Statistical analyses

Analysis was conducted in five steps. First, each ROI was adjusted using regression for variation in intracranial volume (ICV) and age, resulting in standardized Z-scores for each ROI, i.e., the extent to which each subject's values deviated from the expected norm, with mean ± standard deviation at each age = 0 ± 1. Any association with age would indicate an age-disease interaction.

Second, to examine group and country differences, we conducted two separate, 3-group (KS vs. ALC vs. NC) × 2-country (France vs. U.S.) × 2-sex (men vs. women) MANOVAs, one for the four CSF volumes, and one for the two white matter volumes. When these omnibus MANOVAs revealed significant effect of diagnosis, site, and/or diagnosis-by-sites interactions, separate 3-group (KS vs. ALC vs. NC) × 2-country (France vs. U.S.) ANOVAs were conducted, and follow-up tests (Fisher's LSD) were performed for between-group comparisons if necessary.

Third, to address potential influences from scanning parameter differences in the U.S. and France, we re-analyzed the volumetric data using a General Additive Model (GAM) (Hastie and Tibshirani, 1986, 1990; Wood, 2006, 2011) and analysis of variance (ANOVA) from the “mgcv” package in R Version 3.1.0 [http://www.r-project.org/] to test for group diagnostic differences and including covariates of scanner site, sex, age, and supratentorial volume (svol) in the model for each ROI:

$$Model:\;brain_{i}\;\sim \beta_{0}\;+\;\beta_{1}{\text{site}}_{i}\;+\;\beta_{2}{\text{sex}}_{i}\;+\;\beta_{3}{\text{age}}_{i}\;+\;\beta_{4}{\text{svol}}_{i}\;+\;\beta_{5}{\text{diagnosis}}_{i}\;+\;\varepsilon_{i}$$

Fourth, we examined whether alcohol history (i.e., lifetime alcohol consumption, duration of alcoholism, and length of sobriety) and age could explain country-related differences in detected abnormalities. To this end, we carried out bivariate correlation analyses (Pearson) between volumes in each ROI and drinking variables and age in the French and in the U.S. alcoholic groups separately. Family-wise Bonferroni correction for multiple comparisons [*N* = 4 comparisons (three drinking variables and age)/ROI, α = 0.05] was applied with *p* = 0.0125 (one-tailed). Length-of-sobriety data were non-normally distributed and, therefore, log-transformed for correlations. Alcohol history correlations were not conducted in the KS patients because of their unreliable memory.

Fifth, in search of a radiologically-based explanation for the gray matter volume deficits we had observed in our previous analysis of these ALC and KS (Le Berre et al., 2014), we measured volumes of gray matter, white matter, and CSF in the whole brain and sought relations among these measures. The total gray matter, white matter, and CSF volumes were adjusted using regression for variation in intracranial volume (ICV) and age, resulting in standardized Z-scores for each tissue volume. A separate 3-group (KS vs. ALC vs. NC) ANOVA was performed for each tissue volume, and follow-up tests (Fisher's LSD) were applied for between-group comparisons if necessary. In addition, a multiple-regression analysis was conducted to test whether overall shrinkage of white matter or expansion of CSF volumes would selectively predict total gray matter volume.

## Results

**Figure 2:**
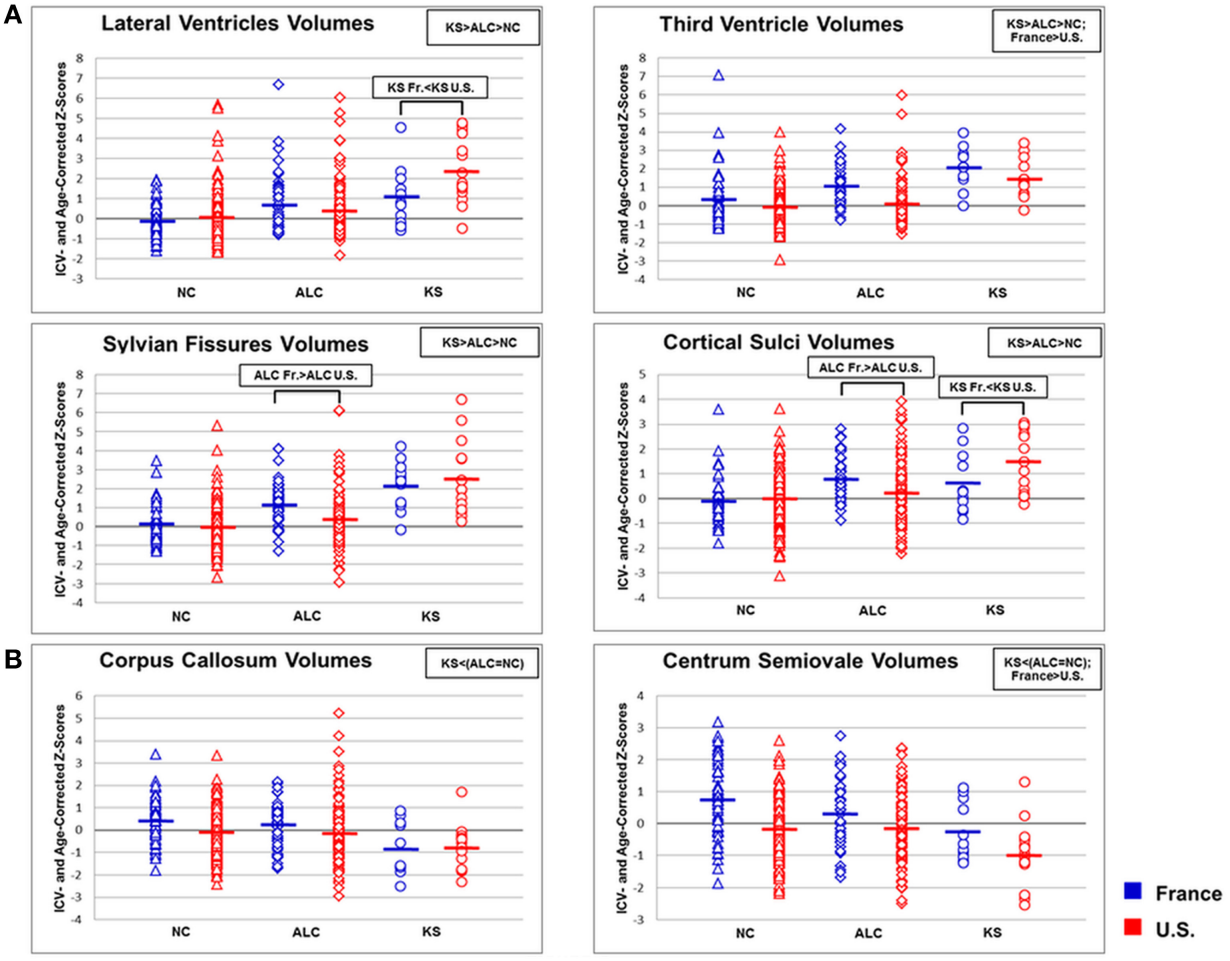
**Scatterplots of (A) brain cerebrospinal fluid (CSF) and (B) white matter volumes in normal controls (NC), alcoholics (ALC), and Korsakoff's Syndrome (KS) patients in France and the U.S**. Means of each column of data are noted by group and by country. Blue dots = France; red dots = U.S.

Statistical results for MANOVAs and ANOVAs conducted on CSF and white matter volumes are presented in Table 3 and Figure 2 and described next.

**Table 3:** **ICV- and Age-corrected Z-Scores (mean ± S.D.) for brain cerebrospinal fluid (CSF) and white matter volumes of the samples from France and the U.S. for the three diagnoses: normal control subjects (NC), patients with alcoholism (ALC), and patients with Korsakoff's Syndrome (KS)**.

		**FRANCE**			**U.S.**			**Group**		**Country**		**Sex**		**Group × Country**		**Group × Sex**		**Country × Sex**		**Group × Country × Sex**	
		**NC**	**ALC**	**KS**	**NC**	**ALC**	**KS**	***F-value***	***p***	***F-value***	***p***	***F-value***	***p***	***F-value***	***p***	***F-value***	***p***	***F-value***	***p***	***F-value***	***p***
**CSF VOLUMES**																					
**MANOVA**		–	–	–	–	–	–	*9.06*	<0.001^*^	*8.48*	<0.001^*^	*1.16*	*ns*	*1.32*	*ns*	*1.43*	*ns*	*0.15*	*ns*	*0.66*	*ns*
**ANOVAs**	Lateral Ventricles	−0.11 ± 0.83	0.71 ± 1.44	1.09 ± 1.49	0.03 ± 1.03	0.39 ± 1.33	2.39 ± 1.62	*29.70*	<0.001^*^ KS > ALC > NC	*4.42*	*ns*	–	–	*5.38*	<0.005^*^ KS U.S.>KS France	–	–	–	–	–	–
	Third Ventricle	0.35 ± 1.42	1.05 ± 1.10	2.06 ± 1.13	−0.08 ± 0.86	0.11 ± 1.12	1.45 ± 0.98	*29.25*	<0.001^*^ KS > ALC > NC	*17.23*	<0.001^*^ France > U.S.	–	–	*2.34*	*Ns*	–	–	–	–	–	–
	Sylvian Fissure	0.17 ± 0.95	1.17 ± 1.11	2.18 ± 1.31	−0.04 ± 1.01	0.40 ± 1.42	2.53 ± 1.92	*47.89*	<0.001^*^ KS > ALC > NC	*1.41*	*ns*	–	–	*3.57*	<0.03^*^ ALC France > ALC U.S.	–	–	–	–	–	–
	Cortical Sulci	−0.10 ± 0.90	0.77 ± 0.85	0.64 ± 1.23	0.02 ± 1.02	0.24 ± 1.33	1.49 ± 1.22	*16.90*	<0.001^*^ KS > ALC > NC	*0.84*	*ns*	–	–	*5.90*	<0.003^*^ ALC France > ALC U.S. KS U.S. > KS France	–	–	–	–	–	–
**WHITE MATTER VOLUMES**																					
**MANOVA**		–	–	–	–	–	–	*4.06*	<0.003^*^	8.50	<0.001^*^	0.10	ns	0.58	Ns	0.95	ns	0.16	ns	0.80	ns
**ANOVAs**	Corpus Callosum	0.45 ± 0.99	0.26 ± 0.99	−0.85 ± 1.19	−0.10 ± 0.97	−0.13 ± 1.36	−0.78 ± 0.98	*9.10*	<0.001^*^ KS < (ALC = NC)	*3.12*	*ns*	–	–	*0.96*	*Ns*	–	–	–	–	–	–
	Centrum Semiovale	0.75 ± 1.21	0.30 ± 1.01	−0.25 ± 0.91	−0.17 ± 0.86	−0.15 ± 1.00	−0.97 ± 0.98	*10.19*	<0.001^*^ KS < (ALC = NC)	*22.74*	<0.001^*^ U.S. < France	–	–	*2.27*	*ns*	–	–	–	–	–	–

* *Significant effect at p < 0.05 in bold and italic*.

### CSF volumes in the three diagnostic groups and two countries

The omnibus 3-group × 2-country × 2-sex MANOVA conducted on the four regional CSF volumes (lateral and third ventricles, Sylvian fissure, and cortical sulci) yielded significant effects of group and country but neither a sex effect nor any interactions (Table 3). Thus, sex was not entered as a covariate in subsequent 3-group × 2-country ANOVAs on volumes conducted on each of the four CSF ROIs (Table 3 and Figure 2).

All four CSF ROIs showed a significant step-wise group effect: KS > ALC > NC. Combining the three diagnostic groups, the total group from France had larger third ventricular volumes than the U.S. group. With the exception of third ventricle, volumes of the remaining three CSF ROIs showed significant group × country interactions, such that Sylvian fissure and cortical sulci volumes were larger in the ALC from France than the U.S., whereas lateral ventricular and cortical sulci volumes were larger in the KS from the U.S. than France (Figure 2A).

### White matter volumes in the three diagnostic groups and two countries

The omnibus 3-group × 2-country × 2-sex MANOVA conducted on white matter volumes showed significant effects of group and country; neither the sex effect nor interactions were significant (Table 3). As this MANOVA analysis did not reveal any significant group × country × sex interactions, sex was not entered into the two subsequent ANOVAs.

Volumes of both the corpus callosum and the centrum semiovale showed significant group effects, indicating that the KS but not the ALC showed volume deficits relative to controls (Figure 2B). A significant country effect for the centrum semiovale volume indicated that participants from the U.S., regardless of group, had smaller volumes than those from France.

### Follow-up analyses accounting for variation in sex, site scanner protocol differences, svol, and age

**Figure 3:**
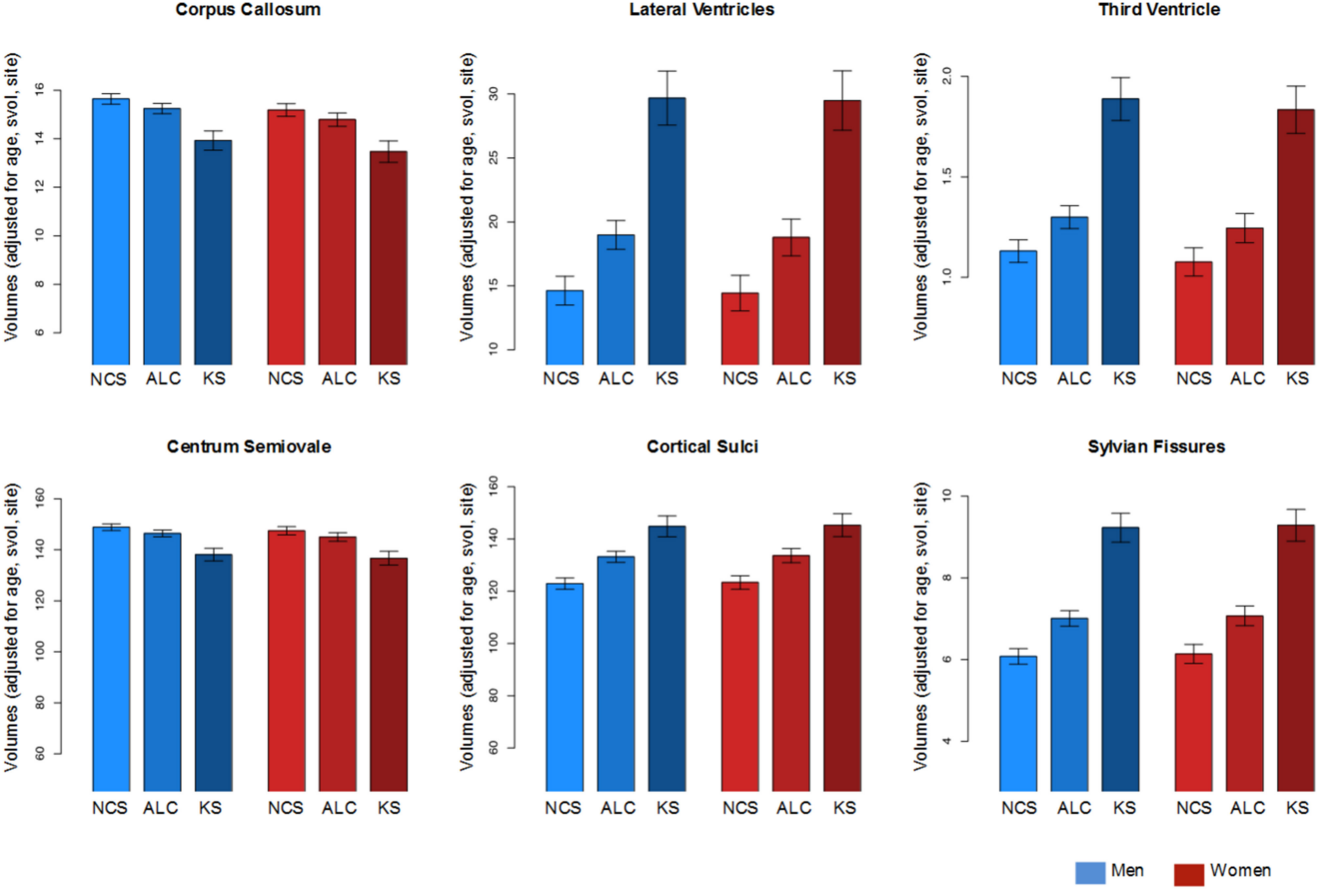
**Mean ± (standard error estimate for each prediction) for each diagnosis and for each ROI**. Men are in blue bars; women are in red bars. Volumes are expressed in cc.

In a follow-up set of analyses, the GAM tested the predictive value of diagnostic group on each brain volume, using country, sex, age, and svol as covariates in the model. From the GAM, a predicted value was derived for each ROI volume for a man or woman of each diagnosis with an svol of 1400 cc, examined at SRI, at age 50 years old (Figure 3). This approach accounts for variation in sex, site scanner protocol differences, svol, and age. Although country, age, and svol (but not sex) made significant contributions to the model predictions, when accounted for, group differences endured for all six ROIs (Figure 3). Specifically, the four CSF volumes showed a graded effect (NC < ALC < KS): Lateral ventricle: ALC *t* = 4.173, KS *t* = 7.437; third ventricle: ALC *t* = 3.211, KS *t* = 7.410; cortical sulci: ALC *t* = 5.226, KS *t* = 5.737; Sylvian fissure: ALC *t* = 5.318, KS *t* = 9.280. The diagnostic effect for the two white matter volumes was prominent for the KS: corpus callosum: ALC *t* = −1.992, KS = −4.458; centrum semiovale: ALC *t* = −1.984, KS = −4.527.

### Alcohol history and age as predictors of CSF and white matter volumes

**Figure 4:**
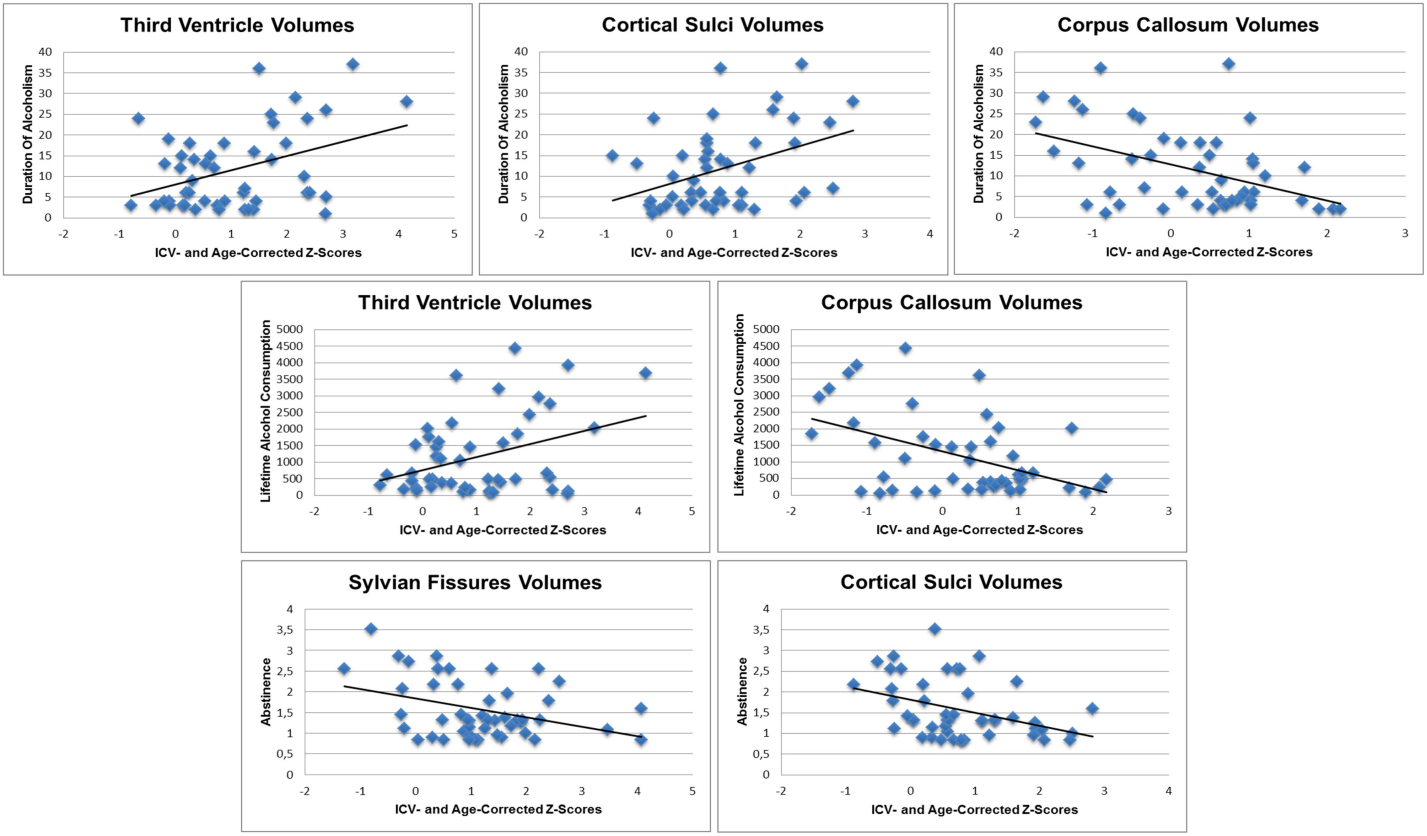
**Scatterplots illustrating the relationships between ROI volumes and their significant alcohol history predictors in French alcoholic patients**.

In the ALC in France but not the U.S., significant (corrected for multiple comparisons) relations emerged: (1) larger volumes of third ventricle (*r* = 0.40; *p* = 0.005) and cortical sulci (*r* = 0.40; *p* = 0.005) and smaller volumes of corpus callosum (*r* = −0.45; *p* = 0.001) correlated with longer duration of alcoholism; (2) larger volumes of the third ventricle (*r* = 0.37; *p* = 0.01) and smaller volumes of corpus callosum (*r* = −0.48; *p* = 0.001) correlated with greater lifetime alcohol consumption; and (3) smaller volumes of the Sylvian fissures (*r* = −0.37; *p* = 0.01) and cortical sulci (*r* = −0.39; *p* = 0.006) correlated with longer sobriety (Figure 4). None of these alcohol history variables predicted volumes of any ROI in the U.S. ALC group. Further, regional age-adjusted volumes did not significantly correlate (*p* = 0.0125) with age in any ROI of either country.

### Regional white mater and CSF volumes as predictors of gray matter volume

**Figure 5:**
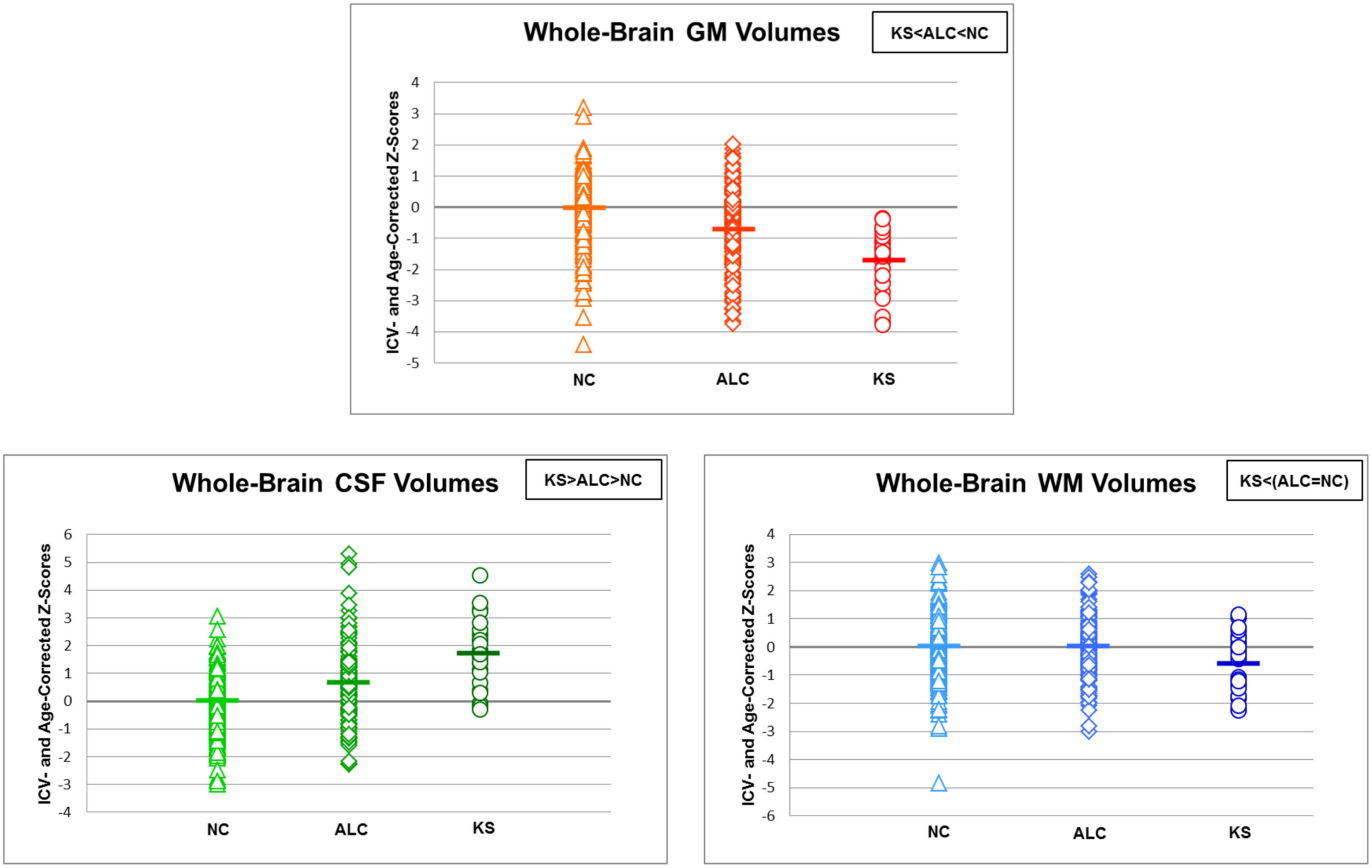
**Scatterplots of whole-brain gray matter, white matter, and cerebrospinal fluid (CSF) volumes in normal controls (NC), alcoholics (ALC), and Korsakoff's Syndrome patients (KS)**. Means of each column of data are noted by group.

First, ANOVAs with follow-up tests (Fisher's LSD) examined group differences in these total brain volumes and yielded step-wise effects for volume of total gray matter (KS < ALC < NC) and total CSF (KS > ALC > NC) but only a total white matter volume deficit in the KS compared with both the ALC and NC, which did not differ from each other (Table 4 and Figure 5). Then multiple regression tested whether shrinkage in white matter or expansion of CSF volumes would selectively predict gray matter volume in the ALC and KS. The overall regression was significant [adjusted *R*^2^ = 0.285; *F*_(2, 188)_ = 38.801, *p* = 0.00000], but the factor that made the greater contribution to the variance of the gray matter volume was CSF volume (*R*^2^ change = 0.277, *p* = 0.00000) compared with a modest contribution from the white matter volume (*R*^2^ change = 0.015, *p* = 0.046).

**Table 4:** **ICV- and Age-corrected Z-scores (mean ± S.D.) for global brain cerebrospinal fluid (CSF), gray matter, and white matter volumes for the three diagnoses: normal control subjects (NC), patients with alcoholism (ALC), and patients with Korsakoff's Syndrome (KS)**.

	**Diagnosis**			**Group**	
	**NC**	**ALC**	**KS**	***F-value***	***p***
CSF volumes	−0.001 ± 1.00	0.66 ± 1.31	1.71 ± 1.20	*38.99*	<0.001^*^ KS > ALC > NC
Gray matter volumes	0.001 ± 1.00	−0.72 ± 1.23	−1.69 ± 0.99	*44.36*	<0.001^*^ KS < ALC < NC
White matter volumes	−0.001 ± 1.00	0.01 ± 1.04	−0.60 ± 1.06	*4.30*	<0.02^*^ KS < (ALC = NC)

* *Significant effect at p < 0.05 in bold and italic*.

## Discussion

The current analysis of alcoholics with and without KS extended our original international report focused on regional gray matter volumes (Le Berre et al., 2014) to examination of regional and global CSF and white matter volumes. Accordingly, our common atlas-based parcellation approach applied to brain images collected in France and the U.S. revealed a graded pattern of brain CSF volume enlargement from uncomplicated alcoholism to KS and white matter volume deficits limited to KS in both nations. The effect sizes of group differences were greatest for the lateral ventricles and Sylvian fissures, thereby indicating these CSF regions as the most sensitive biomarkers of alcohol dependence of those examined herein. CSF volume enlargement and white matter volume deficits correlated with alcohol history variables in the French but not the U.S. alcoholics. Implications of these findings are explicated next.

### Alcohol-related CSF volume enlargement

The rationale for quantifying ventricular and sulcal volumes was three-fold: (1) an inverse relation between CSF and gray matter volumes would support the hypothesis that gray matter shrinkage is associated with an *ex vacuo* process; (2) as a sensitive biomarker of disease (Madsen et al., 2015), CSF space enlargement could enhance detection of factors (e.g., drinking variables, nutrition, type of alcoholic beverage consumed) correlating with, and potentially contributing to, national differences in observed tissue volume shrinkage; and (3) the pattern of observed CSF volume expansion across groups could provide insight into whether uncomplicated and complicated alcoholism reflects a continuum or discrete disorders. Thus we could address the controversy concerning alcoholism as a discontinuous graded effect, which would demarcate discrete differences in disease states between uncomplicated alcoholism and alcoholic KS, or alternatively, characterize alcoholism as a continuum, whereby the distribution of volume measures would be overlapping and ranging from levels of controls to levels of KS (Ryback, 1971; Butters and Brandt, 1985; Pitel et al., 2008, 2012; Sullivan and Pfefferbaum, 2009).

Regarding the first point, we did find support for an *ex vacuo* process in that CSF volumes were highly and inversely correlated with gray matter volumes in alcoholics. That CSF volumes were better than white matter volumes as predictors of gray matter shrinkage is further support for an *ex vacuo* process (Symonds et al., 1999).

Regarding factors contributing to national differences, alcoholics from France but not the U.S. demonstrated relations between brain volumes and alcoholic history variables. Despite having similar levels of lifetime alcohol consumption, the alcoholics from France drank these levels over a shorter duration than alcoholics in the U.S. and implicates national discrepancies in drinking severity profile as contributing to harmful alcohol-related health consequences (Li et al., 2007). Country-related differences in drinking patterns also need to be investigated, such as more or longer phases of abstinence during the alcohol history in the U.S. than in France, or more intermittent heavy drinking episodes (i.e., binge drinking) in the French patients than those in the U.S. potentially contributing to the greater CSF volume abnormalities in France (Hunt, 1993; Squeglia et al., 2012, 2014). Other national differences include the lower legal drinking age in France than the U.S., providing the means for earlier onset, heavy drinking during late adolescence, a time when the brain is continuing development and perhaps especially vulnerable to neurotoxic experiences (Casey et al., 2014; Squeglia et al., 2014). Further, the rather striking differences in the types of alcohol beverages consumed [France: 56% wine, 23% spirits, 19% beer, 2% other; U.S.: 17% wine; 33% spirits, 50% beer (http://www.who.int/substance_abuse/publications/global_alcohol_report/profiles/] may also have an influence on nutrition, in that wine is more likely to be consumed with meals whereas spirits may not be. These factors could accrue and may here have contributed to the relations between drinking history and brain CSF and WM volumes observed in the French but not the U.S. alcoholics. Nevertheless, the hypothesis of exclusive links between brain volumes and alcohol history variables in France compared with the U.S. needs to be qualified as significant correlations were found between gray matter ROIs volumes and drinking history measures in the same French and U.S. alcoholic groups in our previous paper (Le Berre et al., 2014). Especially, in the French alcoholics without KS, significant relationships emerged between greater lifetime alcohol consumption and smaller volumes of lateral frontal cortex, vermis, and pons, and between longer sobriety and larger volumes of lateral frontal cortex and thalamus. In the U.S. alcoholics without KS, greater lifetime alcohol consumption, and shorter duration of alcoholism were each related to smaller thalamic volumes.

Regarding the third point, enlargement in global and regional CSF volumes occurred ostensibly in a graded manner from uncomplicated alcoholism to KS. This graded effect should be considered in the context of national differences. In general, for cortical sulcal and Sylvian fissure volumes, the differences between the alcoholic and KS groups were greater in U.S. than French groups. The smaller uncomplicated alcoholic-to-KS difference in these cortical sulci and fissure volumes in France is consistent with a continuum, but the larger ALC-KS difference in the U.S. is consistent with a discrete, graded effect. To the extent that these differences represent each country in a more general way, these patterns may underlie the longstanding controversy regarding the accurate characterization of alcoholism to KS as a continuum in France (Pitel et al., 2012) and a graded effect (Butters and Brandt, 1985; Sullivan and Pfefferbaum, 2009) in the U.S.

### Alcohol-related white matter volume deficits

Unlike the graded pattern observed in CSF abnormalities, white matter volume deficits were detected only in KS. If white matter abnormalities were present in the current group of alcoholics, they were below detection with MRI. Although white matter volume deficits are known to occur in uncomplicated alcoholics (e.g., Pfefferbaum et al., 1992; Shear et al., 1994; Pfefferbaum et al., 1996; Pitel et al., 2012), they have been shown to be at least partially reversible (Mann et al., 1993; Shear et al., 1994; Pfefferbaum et al., 1995; Cardenas et al., 2007). Consequently, we may have missed the temporal window to observe white matter volume deficits in the alcoholics in our sample; however, this hypothesis was challenged by follow-up correlations demonstrating lack of relationships between length of sobriety and the volumes of the corpus callosum (*r* = −0.01; *p* = 0.91) and the centrum semiovale (*r* = −0.14; *p* = 0.07) in the alcoholic group including French and U. S. ALC patients. Further, several studies have shown diffusion tensor imaging (DTI) to enable detection of white matter microstructural abnormalities in alcoholics who did not exhibit MRI-derived volume deficits (Pfefferbaum and Sullivan, 2002; Pfefferbaum et al., 2014). Those DTI studies implicate myelin degradation, which has been described in postmortem examinations of uncomplicated alcoholics (Lewohl et al., 2000; Mayfield et al., 2002; Harper et al., 2003), a process below detection with bulk tissue measures quantified using structural MRI.

White matter volume deficits occurred in KS from both nations and likely arose from a common cause of severe thiamine deficiency or total thiamine depletion. This likelihood would make KS discontinuous in diagnosis from alcoholics who had not sustained (or did not manifest) such nutritional impoverishment. However, given a heightened vulnerability of chronic alcoholics to sequelae of nutritional deficiencies and metabolic abnormalities (Thomson, 2000; Thomson and Marshall, 2006), in apparently “uncomplicated” alcoholics, sustaining multiple experiences with thiamine or other vitamin deficiencies could lead to compounded effect and a subclinical form of neurological complications resulting in discernable brain structural abnormalities (Pitel et al., 2011).

## Limitations

In light of its retrospective, cross-sectional design, this study has limitations. Based on retrospective data, this study is weakened by a limited access to detailed social, demographic, and clinical descriptive data of the participants groups precluding to investigate potential variables explaining the heterogeneity of cerebral damage in alcoholism. Indeed, factors that would have been desirable to test as potential modifiers of brain volumes are drinking patterns, nutritional history, and medical and clinical information. For example, smoking status and family history of alcoholism can contribute to variability in CSF volumes (Yeh et al., 2007) and other tissue volumes (Cardenas et al., 2005), as can number of withdrawals (O'Daly et al., 2012). Therefore, this retrospective study is a first step of an ongoing international collaborative study between France and the U.S., which includes the recruitment of large cohorts of patients with chronic alcoholism in the two countries with prospective acquisition of neuroimaging, clinical, neuropsychological, biological, and genetic data to identify environmental and genetic factors (pattern of alcohol use, history of withdrawal symptoms including seizures, diet and nutritional status, genes, environment, socioeconomic status, and neuropsychological status) mediating the heterogeneity of brain structural manifestations of alcoholism.

Lack of standardization of clinical instruments and imaging protocols used to collect the imaging or alcohol history data in the different sites is also an inherent limitation associated with retrospective study. For example, alcohol-dependence was determined by DSM-IV criteria in the French group and by either Research Diagnostic Criteria (RDC) for alcoholism (Spitzer et al., 1975) or DSM-IV criteria for alcohol dependence (American Psychiatric Association, 1994) for the U.S. group, depending on inclusion year. Nevertheless, the Research Diagnostic Criteria for alcohol dependence (RDC), a forerunner of DSM criteria, demonstrated a high level of agreement with the DSM-III, with even stricter criteria for determining alcohol dependence than required by DSM (Leonard et al., 1984). Likewise, the lifetime alcohol consumption was quantified through semi-structured interviews, but the questioning was not the same between countries. To mitigate such limitation, the current international multi-site prospective study conducted between France and the U.S. is using similar instruments to derive the alcohol history variables in order to standardize the collection of clinical data between sites and provide more reliable comparisons between countries. Moreover, differences in multisite image data acquisition protocols, such as different slice thickness or orientation, can have a systematic effect on registration accuracy, as well as segmentation, and thus effect region and tissue volume estimates.

Mitigating these factors is the fact that the image data at all sites were T1-weighted SPGR, acquired on the same GE platform at the same magnet strength (1.5T). Further, we took two approaches to address the possibility that the patterns of volume differences among the group were due to differences in acquisition protocols among sites, notably slice thickness or slice orientation. The first approach focused on image processing. Specifically, all images from all four sites were preprocessed and quantified in the aggregate through the same pipeline, which were designed to be unaffected by differences in acquisition protocols; for example, the processing steps make no assumptions about size or orientation parameters. Then, to avoid interpolation artifacts, all regional volumes were computed in the native space of each image, and therefore were based on the exact pixel size and slice thickness of that particular image. A final processing step used a common atlas-based parcellation approach for regional structural identification and volume determination. The second approach focused on data analysis. As a follow-up to the primary multivariate, covariate analysis (MANCOVA), we used a General Additive Model to account statistically for unwanted variance from scanner site protocol differences, sex, age, and supratentorial volume. These analyses yielded the same pattern of group differences identified with the primary analyses. These processing and analysis precautions provide reasonable assurance that the regional brain differences among the diagnostic groups are likely not attributable to image acquisition differences across sites.

## Conclusions

Alcoholism represents a worldwide public health problem. International studies raise awareness of the universality of the personal, physical, and social fallout of this often-intractable disease and enhance medical, dietary, and educational efforts and public policies for care and prevention of alcohol use disorders. Further prospective studies are required to identify sources of heterogeneity of expression and sensitive *in vivo* biomarkers contributing to accurate diagnosis, prognosis, mechanisms of damage, and foundations for targeted treatment.

## Author contributions

Drs. FE, HB, AP, and AB were responsible for MRI data collection at the Caen site. Drs. JM, MR, SC, and CM were responsible for MRI data collection at the Orsay site. Drs. AP and ES were responsible for MRI data collection at the U.S. sites. All authors were responsible for the study concept and design. Drs. TR and AP conducted the image processing and participated with Drs. AB, AP, SC, and ES in data analysis and interpretation of findings. Drs. AB, ES, AP, and TR drafted the manuscript. All authors critically reviewed content and approved the final version for publication. All authors are accountable for all aspects of the work in ensuring that questions related to the accuracy or integrity of any part of the work have been appropriately investigated and resolved.

### Conflict of interest statement

The authors declare that the research was conducted in the absence of any commercial or financial relationships that could be construed as a potential conflict of interest.
